# Advances in bioinformatics and biomedical engineering - special issue of IWBBIO 2013

**DOI:** 10.1186/1742-4682-11-S1-I1

**Published:** 2014-05-07

**Authors:** Francisco M Ortuño, Ignacio Rojas

**Affiliations:** 1 Department of Computer Architecture and Computer Technology, CITIC-UGR, University of Granada, Granada 18071, Spain

## 

In the present issue of Theoretical Biology and Medical Modelling (TBioMed), it is a pleasure to present you a selection of 8 extended versions of selected papers from the International Work-Conference on Bioinformatics and Biomedical Engineering (IWBBIO 2013) held in Granada (Spain) during March 18-20, 2013. IWBBIO 2013 seeks to provide a discussion forum for scientists, engineers, educators and students about the latest ideas and realizations in the foundations, theory, models and applications for interdisciplinary and multidisciplinary research encompassing disciplines of computer science, mathematics, statistics, biology, bioinformatics, and biomedicine. The aims of IWBBIO 2013 is to create a friendly environment that could lead to the establishment or strengthening of scientific collaborations and exchanges among attendees, and therefore, IWBBIO 2013 solicited high-quality original research papers (including significant work-in-progress) on any aspect of Bioinformatics, Biomedicine and Biomedical Engineering.

New computational techniques and methods in machine learning, data mining, text analysis, pattern recognition, data integration, genomics and evolution, next generation sequencing data, protein and RNA structure, protein function and proteomics, medical informatics and translational bioinformatics, computational systems biology, modelling and simulation and their application in the life science domain, biomedicine and biomedical engineering were especially encouraged.

At the end of the submission process of IWBBIO 2013, and after a careful peer review and evaluation process (each submission was reviewed by at least 2 program committee members or additional reviewer), 122 papers were accepted for oral or poster presentation, according to the recommendations of reviewers and the authors' preferences.

A number of authors were invited to submit an extended version of their conference paper to be considered for special publication in this issue of Theoretical Biology and Medical Modelling (TBioMed). These authors were selected after the recommendation of the reviewers of the conference papers, the opinion of the chairs of the different sessions and the guest editors. The extended versions were again carefully reviewed by at least two independent and anonymous experts and the accepted papers, after this new review process, are presented in this issue.

The first paper by Jose J. Castro et al. [1] proposes a new methodology for quantifying and showing night-vision disturbances perceived by subjects, using several experimental conditions. The method is based on the Halo test (freeware software), in which the patient should detect luminous peripheral stimuli around a central high-luminance stimulus over a dark background. In this contribution the influence of the alcohol consumption on visual function is analyzed, observing a relevant deterioration of the discrimination capacity or perception after alcohol consumption.

The paper by J. Zyla et al. [2] has as the main goal the development of a data analysis strategy capable of detecting the genetic background of radiosensitivity in case of studies in which the number of samples is small. In particular, the authors used a sample of 14 inbred mouse strains, being the number of biological replicates between 1 to 3. The methodology proposed in this paper to find candidate polymorphisms, which might be responsible for radiosensitivity phenomena. This method has been carried out using mathematical modelling and data mining techniques simultaneously, using a small sample size. An exhaustive comparative examination of genotypes between the obtained subpopulations of mice followed by functional validation delivers a set of candidate polymorphisms that can be related to radiosensitivity.

The paper titled “A flowgraph model for bladder carcinoma” [3], focuses on bladder cancer. Bladder cancer has motivated numerous contributions in the bibliography, but the evolution of the disease still remains poorly understood. After the tumor has been surgically eliminated, it can reemerge (with similar or even higher level of malignancy). The main goal of this paper is the development and test of a model for the evolution of the disease using a flowgraph model (graphical representation of a multistate model). The proposed model overcomes the disadvantages of other methodologies, such as Markov and semi-Markov processes. The database was obtained from La Fe University Hospital of Valencia (Spain).

In the paper titled “Improvement of Design of a Surgical Interface using an Eye Tracking Device” [4], the authors focused on the development of a novel surgical interface (SI) for kidney tumor cryoablation. The methodology is based on eye tracking technology, that was used in order to improve the design of SI to obtain the optimum configuration, providing quantitative data. This data are used to understand visual and display-based information processing, and the factors that may impact upon the usability of SI. A user-centered design (UCD) approach has been presented for the development of the surgical interface, taking into account the usability and functionality in the design of SI. In order to perform this study, six participants from Department of Faculty of Medicine, Istanbul University used the presented methodology (three of them interacted with the early SI, and another three with the modified SI). The task was to find the tumor on the left kidney (target point), which had been displayed on SI, and to determine a suitable entry point to start the ablation (the participants first took part in a trial practice, during which the participants executed the simple tasks several times to get a basic understanding of the SI). Using NASA-TLX and SPASA questionnaires to measure surgeons’ cognitive load and situation awareness, show that overall mental workload of surgeons related with SI is low.

The paper by Alfredo Benso et al. [5] presents a methodology and a software tool that shows how post-transcriptional regulatory interactions mediated by miRNA can be included in a Boolean Network model (BN), being the starting point the Gene Protein/Product Boolean Network model (GPBN). The proposed tool, supporting an extended BN model, has as main characteristic a more realistic representation of the cell regulatory activity. The proposed software is compatible with visualization tools like Cytoscape and allows running detailed analysis of the network topology as well as of its attractors, trajectories, and state-space. In order to show the performance of the proposed tool, a Gene Regulatory Networks around the mTOR gene is used.

The paper by José Carlos Santos et al. [6] presents a contribution to evaluate if user-generated content could be used to create a reliable predictive model to obtain instant feedback regarding the incidence of flu in Portugal. This model adapts previous studies aiming the prediction of flu incidence by using data from search engine logs or from social media. This proposal is one of the first works on this subject done specifically for the Portuguese language. A novel methodology is presented here based on the combination of tweets and user queries, through multiple linear regression models. In order to evaluate the behavior of the proposed methodology, comparison with epidemiological results from Influenzanet (Influenzanet is a database collected from participants who belong to the project and report any flu symptoms. Over 41200 volunteers from nine European countries, 1552 from Portugal). Using 2704 tweets from Portugal, the authors selected a set of 650 textual features to train a Naïve Bayes classifier to identify tweets mentioning flu or flu-like illness or symptoms, obtaining a precision of 0.78 and an F-measure of 0.83. Using a multiple linear regression model to estimate the data from the Influenzanet project and treating as predictors the relative frequencies obtained from the tweet and query logs classifier, the authors obtained a correlation ratio of 0.89.

In the paper titled “Application of genetic algorithms and constructive neural networks for the analysis of microarray cancer data” [7], the authors presented a comparative study (mainly focused in accuracy performance) of two different feature selection frameworks, namely Stepwise Forward Selection (SFS) and Genetic Algorithms (GA), for the analysis of microarray data. Their main goal was to identify the most significant genes, both for predictive classification purpose and biological relevance. Six standard and machine learning-based techniques (Linear Discriminant Analysis (LDA), Support Vector Machines (SVM), Naive Bayes (NB), C-MANTEC Constructive Neural Network, K-Nearest Neighbors (kNN) and Multilayer perceptron (MLP)) were used, and six free-public cancer datasets were managed to test the several techniques presented. In general, the strategy based on the GA methodology obtain better prediction accuracy index, being these results independent of the classifier used, but it should be noted the high computational complexity of GA framework. Regarding the classifier, C-MANTEC, MLP and LDA showed similar behavior, using a limited set of genes in comparison to SVM, NB and kNN.

Finally, the last selected paper titled “Pathway landscapes and epigenetic regulation in breast cancer and melanoma cell lines” [8] performs a study about breast cancer (BC) and melanoma, analyzing both in parallel on a few cell lines (MCF-7 for BC and A375 for melanoma). The authors begin with a differential expression after treatment with a demethylating agent, the 5-Aza-2'-deoxycytidine (DAC). Results are showed providing pathway enrichment analysis and gene regulatory maps with cross-linked microRNAs and transcription factors. In the first phase, a representative map of the major gene regulators and pathway landscapes involved in the DAC treatment of the MCF7 breast and the A375 melanoma cell lines are obtained. In this contribution 335 DE genes in BC, and 195 DE genes in melanoma upon DAC treatment of MCF-7 and A375 cell lines are identified. Then, a dissection of the key pathways and the knowledge of the interconnectivity between its components have been computed. They are conceived as valuable knowledge to gain towards new therapeutic approaches targeting the common genes involved in oncogenic pathways activated upon DAC treatment.

The Guest Editors would like to express their gratitude to all the contributing authors for their submissions and to the anonymous reviewers for their comments and useful suggestions in order to improve the quality of the papers. They would also like to express their gratitude to Hiroshi Nishiura, Edward Rietman and Rongling Wu, Editors-in-Chief of Theoretical Biology and Medical Modelling (TBioMed), for providing the opportunity to publish this set of selected papers in the present issue. Finally, they are also very thankful to Chantal Stilwell, Assistant Project Manager for Supplements of this journal, for thoroughly helping with the edition and publication of this special issue.

It is a pleasure for us to invite all authors and interested readers of this issue to future IWBBIO conferences, which will be announced at http://iwbbio.ugr.es.

## Competing interests

The authors of this introductory article and guest editors of the special issue declare that they have no competing interests.

## Declarations

Publication of this article has been funded by the Spanish CICYT Project SAF2010-20558

This article has been published as part of *Theoretical Biology and Medical Modelling* Volume 11 Supplement 1, 2014: Selected articles from the 1st International Work-Conference on Bioinformatics and Biomedical Engineering-IWBBIO 2013. The full contents of the supplement are available online at http://www.tbiomed.com/supplements/11/S1.
